# Mouse models of liver cancer: Progress and recommendations

**DOI:** 10.18632/oncotarget.4202

**Published:** 2015-06-05

**Authors:** Li He, De-An Tian, Pei-Yuan Li, Xing-Xing He

**Affiliations:** ^1^ Institute of Liver Diseases, Tongji Hospital, Tongji Medical College, Huazhong University of Science and Technology, Wuhan, China

**Keywords:** hepatocellular carcinoma, animal model, xenograft model, microRNA

## Abstract

To clarify the pathogenesis of hepatocellular carcinoma (HCC) and investigate the effects of potential therapies, a number of mouse models have been developed. Subcutaneous xenograft models are widely used in the past decades. Yet, with the advent of *in vivo* imaging technology, investigators are more and more concerned with the orthotopic models nowadays. Genetically engineered mouse models (GEM) have greatly facilitated studies of gene function in HCC development. Recently, GEM of miR-122 and miR-221 provided new approaches for better understanding of the *in vivo* functions of microRNA in hepatocarcinogenesis. Chemically induced liver tumors in animals share many of the morphological, histogenic, and biochemical features of human HCC. Yet, the complicated and obscure genomic alternation restricts their applications. In this review, we highlight both the frequently used mouse models and some emerging ones with emphasis on their merits or defects, and give advises for investigators to chose a “best-fit” animal model in HCC research.

## INTRODUCTION

Liver cancer is the fifth common malignancy and the second leading cause of cancer-related mortalities in the world [1]. Characterized with high rate of recurrence and metastasis, low detection rate for the curable stages and ineffective therapeutic options, liver cancer, a majority of which is hepatocellular carcinoma, is accepted as a cancer with poor prognosis. Statistics indicate that survival rate of patients after hepatectomy is 30% to 40% at 5 years [2]. Thus, there is an urgent need for research to improve our ability to diagnose, prevent, and treat this disease. Animal models are viewed as crucial tools in the study of liver cancer. Because of the physiologic and genetic similarities between rodents and humans, the short lifespan, the breeding capacity and the variety of manipulating methods, mice are often used for cancer research. While a wide range of liver cancer mouse models are currently available, the universality of each model is limited for several reasons. First, the etiology of liver cancer is rather complicated. Unlike the incidence of lung cancer, which can be reduced by limiting exposure to tobacco smoke, there are many risk factors related with liver cancer, including virus infection [3], chemical carcinogens exposure [4], alcohol abuse [5], or food contaminated with Aspergillus flavus fungus [6]. Different etiologic factors will affect different sets of target genes which result in the genetic heterogeneity of liver cancer and, therefore, make mouse models of liver cancer lack of universality. Second, mouse models can provide researchers with the opportunity to mimic the complex multistep process of liver carcinogenesis, assess tumor–host interactions, perform drug screening, and conduct various therapeutic experiments. However, no model is ideal for all purpose. Each kind of the mouse model can only recapitulate hepatogenesis in some respects. Third, other factors, such as the high requirements on instruments and lack of financial support, also limit the applications of some costly mouse models.

Owing to these facts, a knowledgeable selection should be made according to the specific situation investigators come across in their liver cancer researches. In this review, we assemble and evaluate both the currently used mouse models and the emerging ones related to liver cancer, hoping it would be helpful for researchers to make a choice.

## IMPLANTATION MODELS OF HCC

Characterized by the short modeling period, the relatively lower cost and the suitability in the evaluation of various methods to treat HCC, implantation models, which can be established either by direct implant of tumor tissue fragments or by inoculation of HCC cell lines in recipient mice, have become the most widely used mouse models in current HCC researches [7–12]. According to where the grafts are implanted, implantation models can be divided into two kinds: ectopic models or orthotopic models. Besides, investigators also classify implantation models as allograft models and xenograft models based on whether the grafts and the hosts are from the same species. Since 1969, when the first evidence of human tumor growth in immunodepressed mice has been published [13], human tumor xenografts models have become the priority in preclinical studies. However, in some situations, allograft models may become a better option or even the only option. In this section, we will give out an elaborate description of xenografts models and enumerate some special cases when allograft models are needed.

### Subcutaneous xenograft model

Subcutaneous xenograft model is the most commonly used implantation model in the study of HCC. In this model, human HCC cells or tumor tissue fragments are implanted subcutaneously (usually in the flanks) into immunodeficient mice including nude mice, severe combined immune deficient (SCID) mice or nonobese diabetic-severe combined immunodeficiency disease (NOD/SCID) mice. Subcutaneous xenograft model is often applied to testing the HCC inhibited factors (including new drugs or changes in gene expression). As for new drugs study, administration method could be intraperitoneal, intravenous or intratumoral injection. While intratumoral delivery is of limited value for most tumor types, transcatheter arterial chemoembolization (TACE) recommended by HCC clinical practice guidelines allows directly delivery of drugs or oligos into liver tumor cells. Therefore, intratumoral therapy with tumor suppressor may be feasible for HCC treatment.

Being more easily to access and manipulate, subcutaneous xenograft model established by HCC cell lines are preferred by researches compared with by human tumor tissue fragments. For example, to exam the therapeutic effect of cholesterol-conjugated 2′-O-methyl − modified microRNA-375 mimics (Chol-miR-375), we inoculated 4 × 10^6^ HepG2 cells subcutaneously into the flanks of BALB/c athymic nude mice [14]. When tumor size reached approximately 100 mm^3^, Chol-miR-375 was injected directly into the implanted tumor. By monitored the tumor volume every four days, we demonstrated that Chol-miR-375 can significantly suppress the growth of hepatoma xenografts [14]. Besides, subcutaneous xenograft model may also be used to test the tumor suppressed effect induced by the expression alternation of certain gene. For instance, we once employed 2 × 10^6^ SK-HEP-1 cells which were pre-infected with lentivirus-mediated-anti-miR-221 (miR-221(D)-LV) to establish subcutaneous tumors in nude mice, and found that depletion of miR-221 renders SK-HEP-1 cells less efficient in establishing tumors *in vivo* [15]. Furthermore, we assessed the inhibited effects of miR-221 on subcutaneous xenografts pre-established with 2 × 10^6^ SK-HEP-1 and found that only a single intra-tumor injection with 2 × 10^8^ TU miR-221(D)-LV reduces the growth of tumors [15]. At present, there are many human HCC cell lines which are available commercially. In general, the number of implanted cells is between 10^6^ and 5 × 10^6^ and the tumor formation rate is nearly 100%. As shown in Table 1, we summarized the key information (including the mouse strain, cell lines, cell number, calculation formula and the point to start the therapy) of some frequently used subcutaneous xenograft models in HCC research.

**Table 1:** Subcutaneous xenograft models for HCC

Mouse strain	Cell lines	Total volume	Cell number	Calculation formula	Point to start the therapy	References
Female SCID mice	PLC/PRF/5	200 ul	5 × 10^6^	½W2L	tumor weight = 140 mg–160 mg	[127]
Balb/c nude mice(6–8 weeks)	SK-Hep	100 ul	5 × 10^6^	½W2L	tumor volume = 50 mm^3^	[128]
Balb/c nude mice	HepG2	100 ul	10^6^	½W2L	tumor volume = 200–300 mm^3^	[129]
Female BALB/c nude mice(4–5 weeks)	BEL7404		2 × 10^6^	½W2L	tumor volume = 100–150 mm^3^	[130]
Balb/c nude mice	QGY-7703		10^6^	(π/6)W2L	tumor volume = 100 mm^3^	[131]
Male BALB/C nude mice(4–5 weeks)	HCCLM6	100 ul	3 × 10^6^			[132]
Male NCr athymic mice(5–7 weeks)	Huh7	100 ul	10^6^	0.52 W2L	tumor volume = 200–300 mm^3^	[133]
Male athymic nude mice	PLC/PRF/5	100 ul	10^6^	0.52 W2L	tumor volume = 60–150 mm^3^	[20]
Male BALB/c nude mice	Huh7	100 ul	5 × 10^6^	½W2L	tumor volume = 60–150 mm^3^	[134]
Male BALB/c nude mice	Hep3B	100 ul	5 × 10^6^	½W2L	tumor volume = 60–150 mm^3^	[134]
Female BALB/c nude mice	SMMC-7721		10^7^	½W2L	tumor volume = 100–150 mm^3^	[135]

W, width; L, length.

On the other hand, as tumor tissue fragments retain the characters of human HCC more completely, the results tested on animal models which are established with human tumor tissues may be more reliable. Hu et al. [16] transplanted tumor tissue from each patient to 5 nude mice and assigned them to 5 experimental groups. In their experiment, each group contained 10 nude mice bearing human tumors and represented 10 patients. After the tumor xenografts had grown in size to a diameter of approximately 6–10 mm, they were used to test the antitumor effect of specific Hsp70 expression in cancer tissues combined with cytokine-induced killer (CIK) cells. The results showed that Hsp70 and CIK cells worked synergistically and had a significant inhibitory effect against the growth of HCC xenografts derived from all the 10 patients [16].

Usually, tumor volume (V) is estimated by measuring the length (L) and width (W) with calipers and calculating with the formula V= 1/2(L × W^2^) every a few days. Ge et al. [17], however, monitored the tumor volume by *in vivo* fluorescence imaging system. This method reflects tumor growth more accurately, yet would be more costly at the same time.

Being simple to establish and relatively easy to monitor the size of the tumor, subcutaneous xenograft model is widely used in most experiments aiming to discover some potential tumor suppressed factors. However, its major disadvantage is the lack of interaction between tumors and liver tissues. This is of particular concern because absence of tumor-host relationship may lead to abundance of false-positive responses with drugs. In addition, disruption of microenvironment may affect the biological behavior of malignant cells. For example, spontaneous metastasis rarely occurs when HCC cells are subcutaneously implanted, meanwhile they do metastasize when they are orthotopically implanted [18].

### Orthotopic xenograft model

There are two approaches to establish the orthotopic xenograft model. One is called intrahepatic implantation model. Tumor fragments from patients or subcutaneous xenograft model are cut into 1 × 1 × 1 mm^3^ sized pieces and implanted in the liver of immunodeficient mice [19]. The other, which employed more commonly, is established by injecting tumor cells, always suspended in a volume of 10–20 ul of a serum-free medium containing 50% matrigel, directly into the left hepatic lobe of the mouse [20]. The number of cells needed in orthotopic xenograft model is also about 10^6^ to 5 × 10^6^. And, about one week later, liver tumors will form orthotopically (See Table 2). In some situations, liver tumor in the orthotopic model may metastasis. As the metastasis potential of each HCC cell line is different, the time needed for lung metastases to emerge is distinct. In general, MHCC97H, HCCLM3, and HCCLM6 are accepted as cell lines with high metastatic potential [21]. Yet, by alter the expression level of certain genes, the invasion and lung metastasis capacity of some low metastatic potential cell lines can be promoted. For example, Xia et al. [22] up-regulated Forkhead Box C1 (*FoxC1*) in SMMC7721 cells. Ten weeks after orthotopic implantation, bioluminescence molecular imaging (BLI) showed the presence of lung metastases in the mice implanted with SMMC7721-FoxC1 cells and the absence of metastasis in the control group [22].

**Table 2:** Orthotopic xenograft models for HCC

Mouse strain	Cell lines	Total volume	Cell number	Tumor formation time	References
Athymic nude mice	PLC/PRF/5	20 ul	10^6^	1 wk	[20]
Male BALB/c nude mice	Huh7		2 × 10^6^	1 wk	[134]
Male BALB/c nude mice (6 weeks)	Huh7	50 ul	10^6^		[136]
Male BALB/c nude mice (4-5 weeks)	Hep3B	30–50 ul	2 × 10^6^	2 wk	[23]
Male nude mice	HepG2	25 ul	5 × 10^5^	10 d	[137]
Male nude mice	SMMC7721		5 × 10^5^		[26]
Male BALB/c nude mice	QGY-7703	25 ul	2 × 10^6^		[138]
Male nude mice	HCC97L	30 ul	2 × 10^6^		[139]

The orthotopic xenograft model is superior to the subcutaneous xenograft model in terms of replicating the tumor microenvironment. However, its major defect is that the tumor volume cannot be measured directly unless the mice are sacrificed. To solve this problem, Yao et al. [23] employed the human HCC cell line Hep3B, which is featured by bearing the genome of hepatitis B and producing α-fetoprotein (AFP). In their experiment, they found that AFP was barely detectable in normal mice while increased dramatically and correlated directly with Hep3B tumor growth. Thus, AFP offers a minimally invasive method of monitoring Hep3B tumor progression [23]. In addition, there are also some non-invasive examinations, such as magnetic resonance imaging (MRI) [24] or even positron emission tomography (PET) [25], which can be used for the real-time and non-invasive monitoring of orthotopic HCC progression and metastasis. Optical molecular imaging, including BLI and fluorescence imaging (FLI), is another important technique developed in recent years. In general, tumors can be detected by bioluminescence molecular imaging one week after the orthotopic model has been established [20, 26]. At the same time, it is sensitive to monitor metastases in lung. Being rapid, high-throughput and straightforward as well as easily accessible with much lower instrumentation costs as compared to PET or MRI, optical molecular imaging has been valued as a favorable tool for the study of HCC once it emerged [27]. However, as liver lies in the abdomen, the poor spatial resolution caused by tissue scatter, which means that researchers cannot pinpoint the precise location of the liver tumor, could be a fatal weakness. Fortunately, information about the precise location is not so important in most experiments. These invasive or non-invasive techniques mentioned above make it possible to monitoring the growth of a tumor. Yet, they are either technically complex or instrumentally expensive. Besides, there are some other defects which would also restrict the wide application of orthotopic xenograft model. One is the possibility that inadvertent tumor cells may seed along the needle track or into the bloodstream. The other is that orthotopic xenograft model is technically more challenging compared with the ectopic model.

### Allograft model

In recent decades, accumulating studies reveal that immunotherapy may act as a potentially beneficial option for HCC patients, especially for those who suffer from tumor recurrence [28]. In these cases, immunodeficient mice cannot be used and allograft models which are established by implanting murine HCC cell lines or murine tumor fragments in immunocompetent mice (not necessarily syngeneic) become crucial. As the most appropriate subjects for immunotherapy are patients with relapsed HCC, investigators usually inject tumor cells at a low dose from portal vein [29, 30] or splenic vein [7]. To mimic tumor metastasis, Avella et al. [7] developed an allograft model through seeding of tumorigenic hepatocytes (5 × 10^5^ cells) from Simian Virus 40 T-antigen (SV40 T-Ag) transgenic MTD2 mice into the livers of C57BL/6 mice by intrasplenic injection. Then, they tested the efficacy of sunitinib combined with adoptive transfer of tumor antigen-specific CD8^+^T cells using this model. They found that sunitinib provide immune-enhancing effects by interrupting signal transducer and activator of transcription 3 (STAT3) signaling and regulating the function of distinct immune cells. Sunitinib together with adoptive transfer of tumor antigen-specific CD8^+^ T cells led to elimination of established tumors without recurrence [7]. Hepatic stellate cells (HSCs) have long been considered to contribute to the occurrence and development of HCC. In order to study its immunosuppressive properties, Zhao et al. [31] established a model by injecting a mixture of 1 × 10^6^ mouse hepatoma cells (H22) and 2 × 10^5^ HSCs directly in the liver of BALB/c mice. They found that the activated HSCs promoted HCC growth not only by inducing tumor angiogenesis and lymphangiogenesis, but also by significantly increasing the suppressive immune cell population of regulatory T cells (T_regs_) and myeloid-derived suppressor cells (MDSCs) in the spleen, bone marrow, and tumor tissues of the tumor-bearing mice. This finding provides a new concept in adjuvant immunotherapy. In order to modify the existing orthotopic allograft model to a more reliable one which is accordant to HCC patients with liver fibrosis, Kornek et al. generated liver fibrotic mice by intraperitoneal injection of thioacetamide and oral intake of alcohol. Then, orthotopic allograft model was established in these mice [32, 33]. Subsequently, they found that tumors in fibrotic livers grew significantly larger and more rapidly than those in normal liver. In addition, they even had the capacity to metastasize and form satellite nodules [34].

Allograft model provide us with another choice in some specific research fields. However, as murine liver tumors may differ from human liver tumors, it is doubtful whether the real situation in clinic will agree with those experiment results.

### Xenograft model for liver cancer stem cells (CSCs)-mediated drug resistance

HCC patients even with resection often have a high frequency of recurrence. Unfortunately, systemic chemotherapy is often offered with limited success for those inoperable patients. The reason is that these anticancer therapies mainly kill rapidly growing differentiated tumor cells, thus reducing tumor mass. However, CSCs are resistant to current anti-cancer therapies. They will be left behind and result in relapse of therapy-resistant and more aggressive tumors. For this reason, a mouse model for screening selective agents which can target and eradicate CSCs is needed. To generate such a model, liver CSCs isolated from human HCC fragments or human HCC cell lines [35] will be implanted ectopically or orthotopically in immunodeficient mice. Tumors generated in this way show no major histological differences from the original patient's tumors and, most importantly, drug responsiveness of these tumors better correlates with clinical outcome [36, 37]. This characteristic facilitates the applying of CSCs xenograft models in the liver cancer research. On the other hand, subcutaneous implantation models established by HCC cell lines can also be used in the study of CSCs-mediated drug resistance. Lee et al. [38] establish a HCC nude mouse model with highly chemoresistant MHCCLM3 cells, and treated with either lupeol, large dose of cisplatin and doxorubicin or lupeol plus small dose of cisplatin and doxorubicin. The result showed that lupeol exerted a synergistic effect with low-dose chemotherapeutic drugs. In the following research, they found that lupeol can down-regulate CD133 expression and sensitize HCC cells to chemotherapeutic agents through the phosphatase and tensin homolog (PTEN)–Akt–ABCG2 pathway [38]. The former is a significant marker for hepatic CSCs [39], the latter plays a crucial role in the self-renewal and chemoresistance of tumor-initiating cells [40, 41]. Haraguchi et al. [42] established subcutaneous xenograft models by injecting Huh7 or PLC/PRF/5 into NOD/SCID mice. Then, they demonstrated that combination of a CD13 inhibitor (ubenimex) and the genotoxic chemotherapeutic fluorouracil (5-FU) drastically reduced tumor volume compared with either agent alone. 5-FU inhibited CD90^+^ proliferating CSCs, some of which produce CD13^+^ semiquiescent CSCs, while CD13 inhibition suppressed the self-renewing and tumor-initiating ability of dormant CSCs. Therefore, combining a CD13 inhibitor with a reactive oxygen species (ROS)-inducing chemo/radiation therapy may improve the treatment of liver cancer [42]. The discovery of CSCs-mediated drug resistance brings new vision to the treatment of HCC. Yet, identification and functional characterization of CSCs have been mainly performed in cultured cell lines rather than *in vivo* [43]. Additional validation studies are needed by using animal models, primary tumor specimens and circulating blood cells, which will provide further clinical relevance to support the exploration of CSC knowledge in HCC clinical diagnosis and prognosis.

### GENETICALLY ENGINEERED MOUSE MODELS (GEM) OF LIVER CANCER

Development of transgenic and gene targeting technologies facilitated the generation of GEM to study tumor biology. The most common ways to generate mouse models of cancers are activating oncogenes or inactivating tumor-suppressor genes (or both) *in vivo* through the use of transgenic and gene-targeting approaches. There are numerous works describe the related methods and conclusions in details [44–46]. For this reason, we will explain most of the models briefly and focus on GEM associated with microRNA studies.

### Constitutive gene expression system

Constitutive transgenic mouse is created by transmitting a foreign DNA fragment into a single-cell embryo of the mouse. Thus, when the embryo develops into a mouse, it can express the target gene in all its cells and even transmit the target gene to its offsprings.

Chronic HBV infection is one of the major causes of HCC. The most studied proteins among the compositions of HBV are hepatitis B virus X (HBx) protein and hepatitis B virus surface antigen (HBsAg). Transgenic mice habouring the entire *HBx* gene succumbed to progressive histopathological changes in the liver, beginning with multifocal areas of altered hepatocytes at the age of about 4 months and followed by the appearance of benign adenomas at the age of about 8–10 months. Over 80% males died with hepatocellular carcinoma at between 11 and 15 months while over 60% females at between 17 and 21 months. In this model, AFP can be detected once adenomas formed [47]. HBx protein has been suspected to be a transcriptional transactivator that can stimulate expression of a broad range of proto-oncogenes including *c-fos*, *c-myc* and *c-jun* [48–50], and thus, involved in hepatocarcinogenesis. In addition, HBx protein is found to bind to and inactivates *p53* [51], stimulate the expression of insulin-like growth factor II [52] and the insulin-like growth factor I receptor [53], and compromise DNA repair [54].

On the other hand, transgenic mice with a high expression of HBsAg do develop distinctive inflammation and HCC, especially male mice [55, 56]. When HBsAg is knocked into the *p21* locus, 53.3% male *p21^HBsAg/+^* heterozygotes and 72.7% *p21^HBsAg/HBsAg^* homozygotes developed liver tumors between the ages of 15 and 24 months, yet, no female mice developed tumors at the same ages [55]. In contrast to this model, transgenic mice that overproduce the hepatitis B virus large envelope polypeptide and HBsAg within the hepatocyte developed severe, prolonged hepatocellular injury which processed to neoplasia within 18 months. Consistent with the situation in patients infected with chronic hepatitis B viral, males in the experiment had more tumors than females and at an earlier age [56, 57]. It indicated that HBV acts as a complete carcinogen that causes HCC by initiating a complex series of events in response to chronic hepatocyte injury. Further studies showed that pre-S-1/S-2 mutant HBsAg can induce oxidative DNA damage and mutations in hepatocytes in the late stages of HBV infection and cause hepatocarcinogenesis [58].

Hepatitis C virus (HCV) is also the main cause of HCC worldwide. To demonstrate the chief role of the HCV core protein, Koike et al. [59, 60] generated transgenic mice containing the complete core gene of HCV. These transgenic mice developed hepatic steatosis without inflammation as early as 3 months of age. Then, at 16 months, gross hepatic nodules with the characteristics of hepatocellular adenoma formed. These nodules developed into hepatocellular carcinoma later. Approximately one-fourth of the male transgenic mice had hepatic nodules, while none of the female did (See Table 3). These results were consistent with the epidemiological data that men chronically infected with HCV are more likely to develop to HCC than women [61]. In their further study [62], they found that in core gene transgenic mice over 16 months old the levels of hydroperoxides of phosphatidylcholine was increased by 180%. Interaction of core protein with mitochondria and subsequent oxidation of the glutathione pool and complex I inhibition is an important cause of the oxidative stress seen in chronic hepatitis C [63]. Oxidative Injury would in turn acts as a direct effect of core protein on mitochondria [64] which may contribute to the development of HCC in the absence of inflammation.

**Table 3:** GEM models of liver cancer

System	Transgene	Promotor	Strain	Percentage HCCs	References
Constitutive expression system	*HBx*	HBV	CD1	> 80% in males > 60% in females	[47]
	*HBx + pre C-C sequence*	HBV	C57BL/6xDBA	75% in TG mice at > 15 months	[140]
	*p21 + HBsAg*	HBV	C57BL/6	53.3% in p21^HBsAg/+^males, 72.7% in p21^HBsAg/HBsAg^ males at > 15 months	[55]
	*p21 + HBx*	HBV	C57BL/6	60% in p21^HBx/+^males, 45.4% in p21^HBx/+^females, 63.6% in p21^HBx/HBx^ males and 42.9% in p21^HBx/HBx^females at > 15 months	[55]
	*HCV core, E1, E2*	HBV	C57BL/6	>25.9% in males at >16 months	[59]
Conditional expression system	*HBV*	albumin	C57BL/6 × SJL	100% at 20 months	[56]
	*HCV core, E1, E2*	albumin	C57BL/6 × FVB	100% at 32 weeks (treated with DEN)	[69]
	*c-myc*	albumin	C57BL/6J × CBA/J	65% in males at 20 months	[141]
	*c-myc + E2F1*	albumin	C57BL/6J × CBA/J	100% at 9 months	[142]
	*TGF-α*	metallothionein	CD1	50% in males > 12 months	[70, 143]
	*TGF-α + c-myc*	albumin	C57BL/6J × CBA/J	100% in males at 8 months	[141]
	*SV40 T-antigen*	antithrombin III	C57BL/6 × DBA2	100% at 8 months	[144]
Inducible expression system	*myc*	liver activator protein (LAP)	FVB/N × NMRI	Tumors regress within 3 days after myc inactivation and completely regressed within 30 days	[72]

Constitutive gene expression is technically straightforward and appeared early. However, it has several disadvantages. The major one is the embryonic death phenomenon. Constitutive, especially bilateral expression of some genes may lead to death during embryonic period, which makes it impossible to study the effects of mutations on tumor development in adult mice. Besides, it may induce various types of injury outside of tissues of interest or compensate from related gene products or those in the same pathway [65–67]. Another caveat to this approach is the inability to control the level and pattern of transgene expression on account of the randomness of the transgenic copy number and the integration sites. Random integration of the transgene is of particular concern because it can result in a lack of transgene expression due to positional effects or an unexpected phenotype resulting from secondary effects of transgene integration into sensitive genomic sites. Constitutive transgenic mouse models fail to mimic sporadic multistep tumorigenesis because the initiating mutation is present throughout the body and germ line from the beginning of development. With the advent of conditional and inducible system, investigators have obtained smarter approaches allowing for the induction of somatic mutations in a tissue-specific and time-controlled manner.

### Conditional and inducible gene expression systems

#### Conditional gene expression system

In conditional systems, some liver-specific promoter elements, such as those for albumin [68, 69], metallothionein [70], transthyretin [71], and liver-activating protein (LAP) [72] are utilized. Thus, certain genes can be expressed individually or in combinations only in liver. Sandgren et al. generated a mouse model by directing the expression of *c-myc* to the liver of transgenic mice using the albumin enhancer/promoter [73]. In their experiment, *c-myc* expression can cause mild to severe hepatic dysplasia in young mice, and focal hepatic adenomas in mice over 15 months of age. Further study showed that *c-myc* can activate somatic mutations within the *β-catenin* gene. These alterations lead to a disregulation of the signaling function of *β-catenin* and thus to hepatocarcinogenesis [74]. In addition, to study the interaction between *c-myc* and transforming growth factor α (TGF-α) in hepatic oncogenesis, double transgenic mice bearing fusion genes consisting of mouse albumin (Alb) enhancer/promoter-mouse *c-myc* complementary DNA and mouse metallothionein 1 promoter-human TGF-α complementary DNA was generated [70, 75–78]. Compared with *Alb/c-myc* transgenic mice and TGF-α transgenic mice, time needed to induce dysplastic lesions were reduced significantly. Co-expression of *TGF-α* and *c-myc* began to induce persistent proliferation of the hepatocytes as early as the first weeks of life. This continuous replication lead to neoplastic lesions by the second month of age [75]. At 10 weeks of age, the production of ROS was significantly elevated [79] and a high rate of genomic instability and loss of heterozygosity was observed [80] in *c-myc*/*TGF-α* mice when compared with *c-myc* lesions. Factor et al. [77] even showed that nuclear factor kappa-light-chain-enhancer of activated B cells (NF-κB)-induced survival signaling is activated in preneoplastic and neoplastic lesions of c-myc/*TGF-α* double transgenic mice. However, activation of *β-catenin*, which was most frequent in liver tumors from *c-myc* transgenic mice, was very rare in hepatocellular carcinomas developed in *c-myc*/*TGF-α* mice [81]. It is a shift of profound importance for it provides a general paradigm for characterizing the interaction of nuclear oncogenes/transcription factors and dissecting the genetic and molecular pathways leading to human HCC [75]. Afterwards, *c-myc*/E2 promoter-binding factor 1 (*E2F1*) conditionally transgenic mice [78, 82] and *c-myc*/epidermal growth factor (*EGF*) conditionally transgenic mice [83] were established successively (See table 3). The conditional expression system can circumvents embryonic death effect to a great extent. Yet, it is irreversible strategy and cannot mimic the roles of oncogenes in different stages of tumor progression accurately.

#### Inducible gene expression systems

Inducible system allows for temporal control over genetic changes. Currently, there are three widely used types of inducible systems: (a) tetracycline (Tet)-controlled system (b) tamoxifen-controlled system and (c) virus-mediated Cre delivery system.

**Figure 1:**
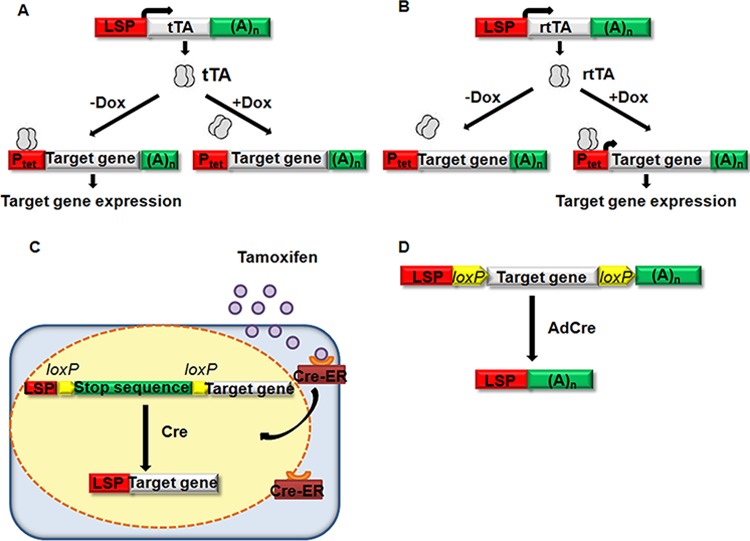
Inducible gene expression system **A.** Tet-Off system, the tet-transactivating protein (rTA), which is driven by LSP, will bind to tet-O promoter sequenes in P_tet_ to activate the expression of a target gene (*myc*, for example) when Dox is not existed. Doxycycline can prevent tTA from binding and abolish the transcription of the target gene [72]. **B.** Tet-On system, the reverse tet-transactivating protein rtTA, which is driven by a LPS, will bind to tet-O promoter sequences and activate transcription of the target gene if doxycycline is existed [86]. **C.** Tamoxifen-controled system, Cre will translocate into nuclear and mediate the recombination of flanked target DNA sequence (stop sequence of HCV, for example) if tamoxifen is present and bind to Cre-ER [88]. **D.** Virus-mediated Cre delivery system, Cre recombinase encoding by adenovirus will inactivate the target gene (*Acp*, for example) which is flanked by loxP sequences [95].

Tetracycline-controlled system consists of two complementary components. One is Tet-Off system, conditionally expressing tetracycline controlled transactivator (tTA); the other is Tet-On system, conditionally expressing reverse tetracycline controlled transactivator (rtTA) (See Figure 1A and 1B). In the Tet-Off system, doxycycline (Dox) prevents binding of tTA to the promoter of Tet (P_tet_), and thus abolishes transcription. By contrast, in the Tet-On system, Dox is needed for rtTA to bind to and activate P_tet_. This inducible system has been used to study liver tumors induced by *c-myc*. Catherine et al. [72] crossed mice with rTA driven by the LAP promoter and myc under the control of the tetracycline-responsive minimal promoter (tet-o-Myc). Thus, progeny possessing both transgenes expressed MYC, whereas mice with either transgene alone or mice with both transgenes and treated with Dox did not express myc. Subsequently, all transgenic mice that overexpressed myc succumbed to liver tumors with a mean latency of tumor onset of 12 weeks. Then, HCC-harboring mice were treated with Dox. Within 4 days, the liver tumors differentiated into normal liver cells accompanied by apoptosis. Within 2 weeks, most of the tumors had grossly regressed. This same system of inducible *myc* expression was used to study the role of microRNA-26a (miRNA-26a) [84] and miRNA-122 [85] in HCC in subsequent studies. MiRNA-26a and miRNA-122 are found to be suppressed in c-myc induced liver tumors. Tet regulatory systems have some favorable properties: (a) it can be set up in a way that allows extremely tight control and, at the same time, regulation over a wide range, (b) Dox concentrations required to quantitatively control expression are far below the toxicity threshold, and (c) Due to the excellent cell and tissue penetration properties of Dox, these concentrations can be readily achieved, even in different compartments of the mouse including the placenta and the milk of lactating mothers. Thus, Tet regulation can be imposed on the developing embryo as well as on the offspring before and during weaning [86].

In the tamoxifen-controlled system, transgenic (tg) mice were produced that express conditionally HCV structural proteins (*core*, *E1*, *E2* and *p7*) in the liver following Cre-mediated DNA recombination. Then, Cre-estrogen receptor fusion protein (Cre-ER) induction strategy was used. As Cre-ER can only response to tamoxifen instead of other endogenous estrogen, Cre nuclear translocation, which led to the expression of HCV protein in the liver, can be induced by intraperitoneal injection of tamoxifen (See Figure 1C) [87–89]. The major disadvantage of this system is the toxicity of tamoxifen and the fusion protein. Excess amounts of tamoxifen can be lethal or can cause damage to certain tissues, such as the uterus [90]. In addition, the Cre-ER fusion protein is toxic in the hematopoietic system [91].

Virus-mediated Cre delivery system is another genetic manipulation. In this system, replication-deficient recombinant adenovirus is commonly used to deliver liver-specific Cre gene to mouse carrying loxP sequence (See Figure 1D) [92, 93]. Colnot et al. constructed a mutant mouse strain in which exon 14 of the tumor-suppressor gene adenomatous polyposis coli (*Apc*) is flanked by loxP sequences (*Apc* ^lox/lox^). By intravenous injection of adenovirus encoding Cre recombinase (AdCre), the *Apc* gene was inactivated in the liver. 8 months after administration of 0.5 × 10^9^ pfu of AdCre, 67% of *Apc* ^lox/lox^ mice developed HCC. β-catenin signaling was found to be strongly activated in these *Apc*-inactivated HCCs [94–96]. Compared with other inducible system, adenovirus-mediated expression system is easier to carry out with a relatively low technical difficulty. Moreover, investigators can achieve not only precise temporal control but also tightly regulation of the expression level of certain gene. However, the adenovirus does not integrate into the host chromosome and thus is unsuitable for stable gene expression.

### GEM involved in HCC-related microRNA (miRNA)

MiRNAs are small, noncoding RNAs, with lengths of 19–25 nucleotides. Recently, numerous researches confirm that liver cancer had an abnormal expression pattern of miRNAs [97]. Modulation of miRNA was considered as a promising therapeutic strategy due to the ability of these small RNAs to potently influence cellular behavior [84]. For these reasons, genetic manipulation animal models for the study of HCC-related miRNA are eagerly awaited. In this section, we will outline several emerging models associated with miRNA.

#### Constitutive and conditional miR-122 knockout mice

MiR-122 is the predominant liver miRNA, making up 70% of the total miRNA population [98]. To elucidate the relevance of miR-122 depletion and HCC development, mutant mice with germ line knockout (KO) or liver-specific knockout (LKO) of the miR-122 locus were generated based on the Cre/loxP recombinase system in two studies [85, 99, 100]. Both mir122-KO (5-week-old) and –LKO (8- to 10-week-old) mice developed microsteatosis and liver inflammation due to triglyceride (TG) accumulation. The microsteatosis progressed to steatohepatitis, fibrosis, and spontaneous HCC. 89% male KO mice and 23% female KO mice developed HCC at the age of 10 months [99], while 50% male LKO mice and 10% female LKO mice did at the age of 12 months. In addition, both KO and LKO mice developed moderately to poorly differentiated AFP-positive HCCs with age. Among the tumor-bearing mice, 3 were also found to have lung metastases. In addition, miR-122 played a protective role against DEN by down-regulating genes involved in proliferation, growth factor signaling, neovascularization, and metastasis [101]. At 35 weeks following DEN exposure, LKO mice exhibited a higher incidence of macroscopic liver tumors (71%) and cysts (86%) compared to a 21.4% and 0% incidence of tumors and cysts, respectively, in control mice. The tumors in LKO mice were bigger and predominantly hepatocellular carcinoma, whereas control mice mostly developed hepatocellular adenoma. DEN treatment also reduced survival of LKO mice compared to control mice. Moreover, miR-122 exhibited tumor suppressor activity when delivered to livers of a non-inflammatory *myc*-driven HCC mouse model [100]. In molecular level, it was confirmed that miR-122 negatively regulates Ccl2 expression by binding to the 3′UTR of Ccl2 mRNA. Without the inhibition of miR-122, Ccl2 expression increased [102]. As a result, the hepatic population of CD11b^hi^Gr1^+^ cells was found to be higher in 10-month-old KO mice than in controls. They produced a high level of IL-6 and TNF-α, which have been shown to promote HCC development by activating the oncogenic transcription factor STAT3 [103]. Besides, several pathways involved in the immune response, fibrogenesis, EMT, signal transduction, cell survival, cell death, and cancer phenotypes, including MAPK, KEGG, PTEN and Akt signalings were significantly enriched. Mir122-KO or LKO mice provide us with a good example to study down-regulated microRNA *in vivo*.

#### A conditionally transgenic mouse model carrying miR-221 gene

Liver-specific miR-221 transgenic mouse is another mouse model targeting on the study of HCC related microRNA. In comparison with wild-type mice, these transgenic mice were characterized by steatohepatitic changes and alternative expression of genes connected to the modulation of the interferon-gamma pathway. After 9 months, about 50% male mice develop small liver tumors. Besides, they also exhibited an increased susceptibility to treatment with the carcinogen [104]. At 6 months, all male animals treated with diethylnitrosamine (DENA) showed evidence of multiple large tumors, whereas in females liver tumors were visible at 9 months. At the molecular level, miR-221 was up-regulated and the targets of miR-221 (*p27, p57*, and *bmf*) were down-regulated in the liver tissue. After *in vivo* delivery of anti-miR-221 oligonucleotides, number and size of tumors were reduced significantly accompanied by a persistent, significant decrease of miR-221 expression [104]. It was the first try to reveal the relationship of HCC and up-regulated miRNA in transgenic mice.

## DIETHYLNITROSAMINE (DEN) INDUCED HEPATOCARCINOGENESIS

Chemically induced hepatocarcinogenesis begin with an irreversible process characterized by structural DNA changes [105]. Based on the carcinogens which increase the instability of DNA, chemically induced hepatocarcinogenesis can be categorized as DEN-induced carcinogenesis, aflatoxine-induced carcinogensis, carbon tetrachloride-induced carcinogenesis, dimethylnitrosamine-induced carcinogenesis and thioacetamide-induced carcinogenesis. Among them, DEN-induced hepatocarcinogenesis model is the most frequently used.

DEN is the hydroxylation of α-hydroxylnitrosamine. After a series of complicated chemical reactions *in vivo*, it turns into an electrophilic agent and reacts with DNA bases to form O6-ethylguanine. Then, to remove the adducts, base excision and the repair of interstrand crosslinking and strand breaks which are considered as major factors in determining DEN carcinogenicity occur [106]. DEN alone is effective to induce liver cancer in mice. In general, 15-days old male C57BL/6 × C3H F1 mice (B6C3F1 mice) are given a single intraperitoneal (i.p.) injection of DEN in a nontoxic dose ranging from 1.25 to 100 ug/g of body weight [107, 108]. Yet, Steffen et al. also induced tumors in 5-week-old male C3H mice by a single i.p. injection of DEN in a dose of 90 ug/g body weight. Tumors will form at least 6 months later. As time goes on, liver tumor incidence can reach 100% [108]. Both the frequency and the time of emergence of lesions is dose-dependent [109]. In addition, DEN-induced tumorigenesis varies with the age, mice strain and sex [110]. The enzymatic competence needed to hydroxylate DEN was already present in newborn animals. It increased and reached peak activity at between the 7th and 15th day of age, and then decreased with age [111]. For this reason, mice less than 15 days of age are more prone to DEN. As to strain differences, male C3H mice are much more sensitive to DEN than C57BL/6 mice. Yet, male C3H mice have the highest rate of spontaneous liver tumors (30–50%) [112], followed by B6C3F1 mice (20–30%) [112] and C57BL/6 mice (less than 2.5%) [113]. Thus, investigators may confuse DEN-induced liver tumors with spontaneous liver tumors. Gender disparity is another interesting phenomenon. HCC occurs mainly in men. Similar sex ratio is also seen in mice given DEN. The incidence of DEN-induced liver tumors is higher in male mice and can be reduced by orchiectomy or gonadotropin blockage; in females the incidence of tumors is increased by ovariectomy or testosterone treatment [114] Naugler et al. found that DEN exposure can promoted production of IL-6 in Kupffer cells (KCs), whereas estrogens can inhibit IL-6 promoter activity by decreasing the activity of the transcription factors nuclear factorkB (NF-kB). As what have mentioned before, IL-6 cause HCC in a manner dependent on signaling via STAT3. Thus, estrogen-mediated inhibition of IL-6 production reduces liver cancer risk in females. In addition, DEN-induced tumorigenesis is dose-dependent and even varies with the age, sex and mice strain [110]. Carcinogenicity of DEN is enhanced by phenobarbital (PB) [115] and by the expression of *foxm1b* [116], hepatocyte growth factor (HGF) [117], Interleukin-22 (IL-22) [118] or macrophage inflammatory protein-1 and its receptor (CCR1/CCL3) [119]. Meanwhile, curcumin [120], suppressor of cytokine signaling 3 (*socs3*) [121] and *ptpn11* [122] are reported to act as tumor suppressors in the carcinogenicity of DEN.

Regardless of the dose, sequential histochemical changes, from basophilic foci to hyperplastic nodules, then followed by hepatocellular adenomas, and finally hepatocellular carcinomas, will occurred during the hepatocarcinogenesis process induce by DEN [110]. In this process, the levels of fat and glycogen and the activities of several enzymes involved in cell membrane function, glycogen metabolism, the oxidative pentose phosphate pathway, and glycolysis [107]. All the changes are closely related to the neoplastic conversion of hepatocytes [123]. Comparative functional genomics showed that the gene expression patterns in HCCs in diethylnitrosamine-induced mouse liver cancers were most similar to those of the poorer survival group of human HCCs [124]. However, there are some problems concerning on this model. Though employing a various kind of methods to accelerate hepatocarcinogenesis, the average time for forming a tumor is still quite long. Besides, the temporal and spatial heterogeneity of tumor formation also bother the investigators. These shortcomings limit its usage severely.

## SPONTANEOUS ANIMAL MODELS OF HCC

Due to different genetic background and numerous other factors, mouse strains differ in susceptibility to spontaneous hepatocellular neoplasia. In general, C3H mice and CBA mice are recognized as being prone to spontaneous liver tumors, while LP, 129, DBA/2, BALB/c, and C57BL are mouse strains with low incidence of spontaneous hepatocellular neoplasia. However, the specific incidence rate for each strain, which has been documented in the published literature since the late 1930's and early 1940's, could hardly be captured now [125]. The fatty liver Shionogi (FLS) mouse is an inbred strain that develops spontaneous fatty liver (hepatic steatosis) chronically without obesity. Masahiko et al. reported that the mice develop inflammatory, steatohepatitis and spontaneous hepatocellular tumors. An incidence of HCC was 40% in males at 15–16 months of age, while it was 0% at 13–16 months and 9.5% at 20–24 months in females [126]. Yet, with an extremely low incidence rate, the awfully long time needed to establish the model, and the heterogeneity of tumorigenesis, spontaneous HCC models are considered as an uncontrollable and unpredicted system, and therefore, are scarcely used nowadays.

## CONCLUSION

An ideal animal model should faithfully reproduce the key biological behaviors of liver cancer, adequately mimic the human tumor microenvironment, be affordable and easy to manipulate [44]. However, ideal animal models for liver cancer research are unavailable to date. Fortunately, all of the existing liver cancer models can be regarded as valuable and predictive tools if used appropriately and taking the limits of these models into account. Usually, GEM are excellent models for studies concerning on the molecular mechanism of liver cancer development; with a relatively higher metastasis rate, orthotopic HCC models are appropriate for HCC metastasis investigations; both subcutaneous and orthotopic models could be applied to test new therapeutic strategies. With the development of genome editing techniques and the spread of valuable instruments, mice models of high quality will be more accessible to investigators and, doubtlessly, improve our understanding of liver carcinogenesis and the design of comprehensive treatment strategies.

## Abbreviation

HCC: hepatocellular carcinoma
GEM: genetically engineered mouse models
miR: microRNA
CSCs: cancer stem cells
DEN: diethylnitrosamine
HBV: hepatitis B virus
HCV: hepatitis C virus
SCID: severe combined immune deficient
NOD/SCID: nonobese diabetic-severe combined immunodeficiency disease mice
TACE: transcatheter arterial chemoembolization
Chol-miR-375: cholesterol-conjugated 2′-O-methyl − modified microRNA-375 mimics
AEG-1: astrocyte elevated gene-1
FoxC1: forkhead box c1
BLI: bioluminescence molecular imaging
AFP: α-fetoprotein
MRI: magnetic resonance imaging
PET: positron emission tomography
FLI: fluorescence imaging
SV40 T-Ag: Simian Virus 40 T-antigen
STAT3: signal transducer and activator of transcription 3
HSCs: hepatic stellate cells
T_regs_: regulatory T cells
MDSCs: myeloid-derived suppressor cells
PTEN: phosphatase and tensin homolog
5-FU: fluorouracil
ROS: reactive oxygen species
HBx: hepatitis B virus X protein
HBsAg: hepatitis B virus surface antigen
HCV: hepatitis C virus
LAP: liver-activating protein
TGF-α: transforming growth factor α
Alb: albumin
E2F1: E2 promoter-binding factor 1
EGF: epidermal growth factor
tTA: tetracycline controlled transactivator
rtTA: reverse tetracycline controlled transactivator
Tet: tetracycline
P_tet_: the promoter of Tet
Dox: doxycycline
tet-o-Myc: tetracycline-responsive minimal promoter
Cre-ER: Cre-estrogen receptor fusion protein
Apc: adenomatous polyposis coli
AdCre: adenovirus encoding Cre recombinase
TG: triglyceride
KO: knockout
LKO: liver-specific knockout
PB: phenobarbital
IL-22: Interleukin-22
HGF: hepatocyte growth factor
FLS: fatty liver Shionogi
